# The DIY IVF cycle—harnessing the power of deeptech to bring ART to the masses

**DOI:** 10.1007/s10815-022-02691-x

**Published:** 2022-12-14

**Authors:** Lynae M. Brayboy, Alexander M. Quaas

**Affiliations:** 1 Department of Neuropediatrics, Charité-Universitätsmedizin Berlin, Berlin, Germany; 2 Clue By Biowink, Berlin, Germany; 3 The Bedford Research Foundation, Bedford, MA USA; 4 Division of Reproductive Medicine and Gynecologic Endocrinology, University Hospital Basel, Frauenklinik Vogesenstrasse 134, 4031 Basel, Switzerland

**Keywords:** In vitro fertilization, Assisted reproductive technology, Digital health, Deeptech, Femtech, Wearables, Remote monitoring, Health technology

## Abstract

The emergence of telehealth including telemedicine, at-home testing, and mobile health applications has enabled patients to self-manage their reproductive care, especially during the COVID-19 pandemic. Reproduction is rapidly changing and embracing deeptech initiatives that can improve outcomes and facilitate personalized fertility solutions in the near future. This so-called DIY IVF informed by deeptech and moderated by femtech not only holds a tremendous amount of promise, but also challenges and possible pitfalls. This review discusses the current status of deeptech and femtech for IVF care in a post-Roe v. Wade environment.

Globally, as many as 186 million people suffer from the disease of infertility, a major public health problem [1]. In the USA, it is estimated to affect 6.7 million women, yet assisted reproductive technology (ART) remains inaccessible for the majority of the US and the world’s population [2]. One of the largest hurdles to reproductive health equity and access is the very high cost of fertility care [3].

Efforts to make treatment cycles more affordable are underway in the UK and some European countries, which has led to an expansion in treatment numbers [4].

However, many barriers to access reproductive services still exist. Therefore, many couples have turned to inexpensive and less constrained alternatives such as seeking care abroad via “reproductive tourism” [5]. In the setting of donor insemination, women may turn to unscreened and unregulated sperm donors for financial reasons [6]. In addition to the cost, the global Coronavirus pandemic, restrictions, and invasive nature of ART, with frequent transvaginal ultrasounds and on-demand sperm production, have driven many couples seeking reproductive care to in-home alternatives that utilize health technology or “telehealth” [7]. According to the American College of Obstetricians and Gynecologists (ACOG), telehealth encompasses all technology-enhanced healthcare frameworks that include services such as virtual visits, remote patient monitoring, and mobile (smartphone)-based healthcare. As defined in ACOG Committee Opinion Number 798 “Implementing Telehealth in Practice”, the word *mHealth* specifically refers to smartphone applications that permit self-managed patient care using mobile phones or other wireless technology and does not necessarily involve monitoring by a physician [8]. There are more than 100,000 mHealth apps available [9] with more than 3.7 billion downloads globally [10]. Since mHealth apps were introduced in 2008, they have had a massive impact on daily life. It has been estimated that more than 500 million smartphone owners use some form of healthcare app [11]. Prime examples of mHealth applications in reproduction include period trackers. In the context of fertility care, mHealth apps may be used in a variety of ways. Menstrual cycle mHealth apps or period trackers may be used to enhance reproductive health literacy in couples trying to conceive, or prevent pregnancy in women as a form of digital contraception. Digital contraception using period tracking mHealth apps is highly controversial as the vast majority are unregulated and are not evidence-based. There are only two FDA-cleared “software as a medical device (SaMD)” digital algorithms available on mHealth apps that simultaneously support conception and contraception via technology-enhanced digital fertility awareness-based methods on the market: Natural cycles, which uses a basal body temperature-based algorithm that requires a thermometer or a temperature checking wearable and Clue Birth Control and Clue Conceive, an algorithm-based method which uses the menses as a biomarker and accounts for cycle variability to give predictions about high and low-risk days throughout the menstrual cycle. Clue Birth Control/Clue Conceive, formerly the Dynamic Optimal Timing (Dot) algorithm [12], does not require hardware or wearables. Healthcare providers are just beginning to understand how their patients may be utilizing these tools to manage their reproductive needs, but more data is needed to determine how and if mHealth apps improve reproductive outcomes among users.

## A deeptech revolution powered by Femtech

Just as machine learning is a subset of artificial intelligence (AI), femtech innovations will power the deeptech revolution in ART. The industry term “femtech” was coined by Ida Tin, the Danish-born founder of a period and ovulation tracking mHealth app established in Germany in 2013 [13]. Femtech refers to all technology that relates to electronic devices, software, and other technology relating to women’s health. Femtech is a growing field that will provide the innovations to power the deeptech revolution for ART, with a projected multibillion-dollar market: in 2019, the femtech industry attracted $592 million in total venture capital investment, and generated $820.6 million in global revenue [14].

The term deeptech refers to problem solving for fundamental issues that present substantial engineering challenges. Examples of companies that do this include Moderna, SpaceX, and Blue Origin. Deeptech companies tend to have physical products and patents that translate the power of “big data” and computational analytics to solve problems in the “real world.” Within ART, an illustrative example is the company TMRW. It is a robotics and automation of cryogenic sample management with a software solutions platform that integrates with the identification of patients and medical records, and EMR systems. The greatest problem ART faces is its high costs, yet consistently low (less than 50%) success rates. In addition, ART multiple fetal gestations, gynecologic, and obstetric complications are other major strains on the healthcare economy. However, AI is poised to lower healthcare costs because of automation and streamlined workflows that can improve the various proposals for measuring IVF costs, e.g., dollars per baby, time to baby, life disruption to baby, clinic workflow, laboratory workflow, and process optimization as possible outcome metrics.

The long-term goal of AI, automation, innovation, and digitalization is that IVF could become more accessible to the wider public. AI democratizes IVF by removing the need for high-overhead, high-complexity embryology laboratories and leverages fewer, highly skilled staff to oversee greater numbers of IVF cycles without sacrificing quality and safety. Labor is the major cost in an IVF center, and there is a worldwide shortage of expert embryologists and board-certified reproductive endocrinologists. Although the USA trains and certifies the majority of the world’s REIs, with approximately 50 American Board of Obstetrics and Gynecology certified programs nationally [15], the majority of Americans do not have access to IVF care [16].

Predictive modeling, the basis for AI technology (e.g., taking young age, long menstrual cycles, polycystic ovary syndrome (PCOS), anti-Müllerian hormone (AMH), and antral follicle count (AFC) into account) has already been shown to reduce cycle cancellation and patient hospitalization for ovarian hyperstimulation syndrome (OHSS), achieving a significant reduction in costs [17]. Another example of cost reduction is with PGT. The most reported disadvantages of PGT-A were increased cost, clinical uncertainty about how to handle mosaic results, and concerns that the trophectoderm biopsy is not representative of the entire embryo. AI can potentially answer these huge challenges in assisted reproduction. Furthermore, embryo selection via AI will facilitate reduced costs through the optimization of laboratory performance, shorter time to a healthy, live birth of a singleton, and reduction of failed cycles through avoidance of transferring embryos with a low chance of implantation (deselected embryos). There will also be cost savings associated with the reduction in manual labor staff hours and consumables used. As AI systems increase in accuracy, we will continuously uncover new determinants of IVF success, the predictive ability will grow, continuing to minimize costs and patient drop-out due to financial treatment fatigue.

## Telemedicine, apps, and wearables

External hardware add-ons may allow users to perform enzyme-linked immunosorbent assay (ELISA) assays with the help of a 3D-printed opto-mechanical attachment to hold and illuminate a 96-well plate using a light-emitting-diode (LED) array [18]. Similar add-ons exist for microscopy and even ultrasonography [19]. The large amounts of data collected by the users of femtech apps may be synthesized into large real-world studies [20–22] and could serve as starting points for large-scale genomic and metabolomic studies designed to understand the various etiologies of infertility. Smartphone application manufacturers are already partnering with academic institutions to conduct long-term studies on gynecologic conditions [20, 23]. Boosted by the COVID-19 pandemic, numerous telemedicine fertility platforms such as Apricity have flourished to provide virtual consultations for fertility care and offer AI-based fertility predictors based on patient characteristics. Appropriate use of telemedicine is crucial given the risk of missing conditions without in-person care abetted by physical exam. In selected low-risk patients, the potential benefits outweigh the risks. In theory, a patient could be seen for the initial consultation via telemedicine appointment and then submit test results and other information through smartphone apps and wearable devices, never actually entering the clinic until a procedure is required.

## The DIY IVF cycle

How can clinicians reliably harness and use the information and diagnostics that these new tools provide? How can a clinic seamlessly integrate apps and platforms into its workflow and electronic health record (EHR)? How will patients feel about the increasing digitalization and automation of their care? Maybe in the not-too-distant future, patients will have an external ultrasound and blood testing hardware add-ons, allowing for automated 3-D follicular scans and blood draws at home. The data would be transmitted to the clinic and analyzed through AI. Analyzing information from thousands of cycles to train “machine learning” algorithms has displayed promise for individualized gonadotropin dose adjustments, which could be implemented instantly via a subcutaneous medication administration system similar to an insulin pump [24].

What are the advantages and pitfalls of this “brave new fertility world”?

The “self-service IVF cycle” may result in a reduction in staff costs for the clinic, which may reduce treatment costs for patients and democratize access. It could also make fertility treatment more convenient for busy professionals with little time to attend clinic appointments. Telemedicine consults with lab results could assist clinics in triaging patients who might need more immediate IVF versus those who qualify for more conservative management.

However, telemedicine does reduce human interaction, an intangible factor that should not be underestimated. In supermarkets with an automated checkout option, a customer may still choose to go to the cashier to get a warm greeting and human interaction. Likewise, the regular interaction with an empathetic doctor may provide emotional strength for a patient during the rollercoaster journey of an ART cycle.

An additional concern regarding the use of information technology (IT) and artificial intelligence (AI) in ART is the topic of data security. Undergoing fertility treatment is a highly intimate and private experience, and the consequences of unintended or intended data breaches are enormous. Akin to the banking and customer service industry, every fertility clinic using IT and AI as part of the treatment will need a dedicated cybersecurity strategy. It is also important that best practice industry standards for all femtech that can be used to evaluate not only the data privacy, but also the regulation and quality of the technology. Some consideration should also be given to inclusivity and diverse populations being included in the resulting databases and machine learning/AI so that algorithms are representative. The promise of all these gadgets and innovations, and the human perils (bias, emotional, financial, security), will only be realized and mitigated through the application of deeptech. Deeptech is needed to integrate these technologies and approaches to accelerate and redefine fertility innovation for decades to come.

## Data privacy in the era of the reversal of Roe v. Wade

With the recent United States Supreme Court decision, Dobbs vs. Jackson, data privacy became a top priority overnight given the fear of reproductive surveillance. Individual states that have banned abortion have crafted unique and unusual laws to coordinate surveillance of women and allow citizens to become de facto bounty hunters [25]. Citizens of such states are incentivized to sue clinics, doctors, nurses, and even people who drive a woman to get the procedure, for financial gain. Social media and media influencers are calling on women to delete their period and fertility trackers for fear of reproductive surveillance. Articles regarding data privacy and period tracking have been published in the New York Times [26] and Washington Post (27). This has even caught the attention of a Congressional oversight committee that called on period and fertility tracking applications to submit their data privacy policies. There is currently a bill proposed by Rep. Sarah Jacobs D-California that would limit reproductive data collection and provide stricter stipulations regarding consent. Regardless of the data repository, be it a mHealth application or an EHR, it is incumbent that strong patient/customer privacy protections prevail to ensure data-driven technology. It remains to be seen how this climate will help or hinder patients from utilizing period tracking or software as a medical device to prepare for home testing or home inseminations in the USA.

## The future

AI is likely to change every aspect of ART as we know it: the laboratory by aiding embryo and gamete selection, the clinic by tailoring the treatment protocol to an individual patient based on their lab testing and diagnoses or matching a donor to a recipient using tools such as facial recognition software; and through the advent of the “DIY IVF cycle.”

The promise that these innovations carry for providers and patients is massive.

Deeptech may truly help to bring ART to the masses. Likewise, great risks are inherent to this rapid development—some of which are obviously apparent, and some still to be discovered. With the implementation of each new technology, it is vital to ensure that the cost of treatment will ultimately be reduced by it and not increased, with the opposite effect on access to care and live birth rates (which should remain, at a minimum, non-inferior).

The art of medicine is a human endeavor, and increasing digitalization and automation should not alienate patients from providers, increase inequality or undesirable outcomes. This special issue of JARG takes a look at how a great temblor of deeptech is taking shape in reproduction and fertility. As technological advances move from the research lab to the IVF clinic and marketplace, and as companies form to pursue commercial applications, we see a number of similarities in how and why they are being developed—and a powerful ecosystem is taking shape to drive their development. For deeptech ventures to be successful in reproduction, they will need to bring together multiple talents (including embryologists, scientists, fertility physicians, engineers, and entrepreneurs) to solve these complex problems. As attractive as every new technology appears to the majority of scientific-minded people with a love for gadgets, we should not lose track of our primary goal: helping patients overcome the disease of infertility as economically and painlessly as possible.
